# Microbial community structures differentiated in a single-chamber air-cathode microbial fuel cell fueled with rice straw hydrolysate

**DOI:** 10.1186/1754-6834-7-9

**Published:** 2014-01-17

**Authors:** Zejie Wang, Taekwon Lee, Bongsu Lim, Chansoo Choi, Joonhong Park

**Affiliations:** 1 Department of Environmental Engineering, Daejeon University, Daejeon, South Korea; 2 Department of Applied Chemistry, Daejeon University, Daejeon, South Korea; 3 School of Civil and Environmental Engineering, Yonsei University, Seoul, South Korea; 4 Institute of Urban Environment, Chinese Academy of Sciences, Urban, China; 5 Department of Microbiology and Ecosystem Science, University of Vienna, Vienna, Austria

**Keywords:** Microbial fuel cell, Microbial diversity, 454-pyrosequencing, Rice straw biomass

## Abstract

**Background:**

The microbial fuel cell represents a novel technology to simultaneously generate electric power and treat wastewater. Both pure organic matter and real wastewater can be used as fuel to generate electric power and the substrate type can influence the microbial community structure. In the present study, rice straw, an important feedstock source in the world, was used as fuel after pretreatment with diluted acid method for a microbial fuel cell to obtain electric power. Moreover, the microbial community structures of anodic and cathodic biofilm and planktonic culturewere analyzed and compared to reveal the effect of niche on microbial community structure.

**Results:**

The microbial fuel cell produced a maximum power density of 137.6 ± 15.5 mW/m^2^ at a COD concentration of 400 mg/L, which was further increased to 293.33 ± 7.89 mW/m^2^ through adjusting the electrolyte conductivity from 5.6 mS/cm to 17 mS/cm. Microbial community analysis showed reduction of the microbial diversities of the anodic biofilm and planktonic culture, whereas diversity of the cathodic biofilm was increased. Planktonic microbial communities were clustered closer to the anodic microbial communities compared to the cathodic biofilm. The differentiation in microbial community structure of the samples was caused by minor portion of the genus. The three samples shared the same predominant phylum of *Proteobacteria.* The abundance of exoelectrogenic genus was increased with *Desulfobulbus* as the shared most abundant genus; while the most abundant exoelectrogenic genus of *Clostridium* in the inoculum was reduced. Sulfate reducing bacteria accounted for large relative abundance in all the samples, whereas the relative abundance varied in different samples.

**Conclusion:**

The results demonstrated that rice straw hydrolysate can be used as fuel for microbial fuel cells; microbial community structure differentiated depending on niches after microbial fuel cell operation; exoelectrogens were enriched; sulfate from rice straw hydrolysate might be responsible for the large relative abundance of sulfate reducing bacteria.

## Background

Microbial fuel cells (MFCs) are devices to produce electric energy from organic matters and treat wastewaters in both anode and cathode chambers [1,2]. Pure organic compound, real wastewater, and biomass have been successfully used as fuel for power generation in MFCs [3]. Rice straw is one of the most abundant biomasses, mainly composed of cellulose, hemicellulose, and some lignin [4]. The hemicellulose can be easily degraded to its constituent sugars through acidic and/or enzymatic hydrolysis; the produced sugars can be further used as substrate to produce organic acids or bioethanol [5,6]. Therefore, the sugars produced from the rice straw hydrolysate might be used as a useful fuel for power generation from MFCs.

In MFCs, microbes play crucial roles in energy output and organic contaminants removal [7]. The ability of microbes to transfer electrons in the anode can significantly affect the performance of MFCs. Anodic microbial communities were reported to be significantly related with the types of substrates fed into the anode chamber [8]. For example, *Acetobacterium* species (sp.), *Geobacter* sp., and *Arcobacter* sp. were detected in the anodic biofilm fed with formate [9]; however, *Enterobacter* sp. was the dominant bacterial species in the MFC with glucose as substrate [10]. For air-cathode MFCs, biofilm was commonly formed on the water-facing side of the cathode. It was discovered that the formation of biofilm on the Pt-loaded air-cathode could decrease the power output due to the increased cathodic resistance and limited proton transfer rate [11]; however, recent research demonstrated that the biofilm formation on a bare air-cathode could enhance the electric power output from air-cathode MFCs [12]. The different research conclusions might be caused by different air-cathode configurations. Moreover, the cathodic biofilm in a Pt-loaded air-cathode was observed to be capable of removing nitrogen, with enhanced removal efficiency due to the pre-accumulation of nitrifying biofilm [13]. The aforementioned results indicate that the cathodic biofilm deserves further research.

Therefore, the purpose of the present study was to evaluate the availability of diluted acid-treated rice straw hydrolystate as fuel for an air-cathode MFC. In addition, microbial analysis at high resolution level using 454 pyrosequencing was carried out to evaluate the effect of the rice straw hydrolystate and niches on the microbial diversity and community.

## Results and discussion

### Performance of the MFC

**Figure 1:**
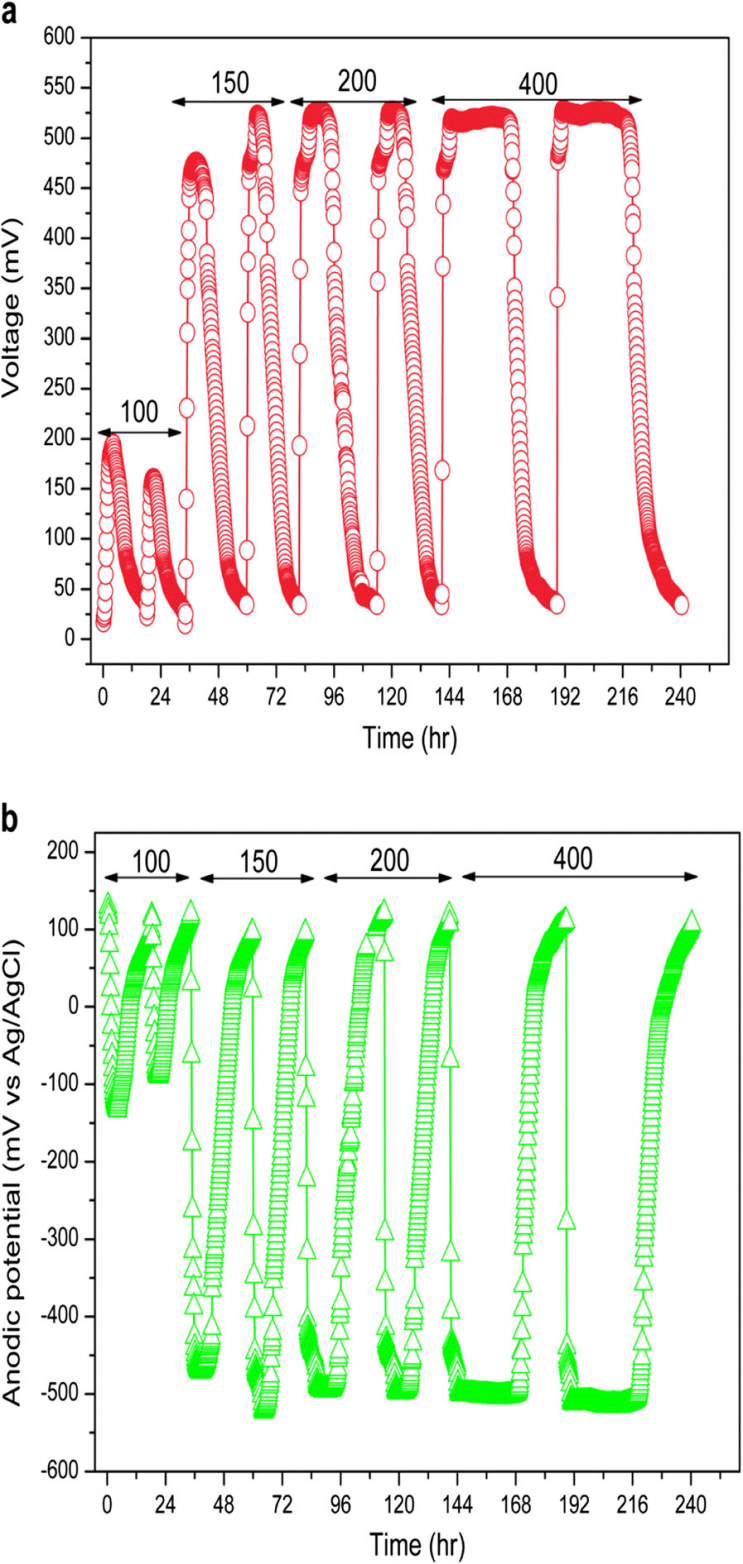
**Voltage response of the microbial fuel cell (MFC) to chemical oxygen demand (COD) under batch-mode operation. (a)** Voltage output and **(b)** anodic potential as a function of COD concentration at a fixed external resistance of 500 Ω. Figures on the plot represent the COD concentrations (mg/L).

After addition of the rice straw hydrolysate as an anodic solution, cell voltage was immediately increased with no lag time. Stable voltage increased from 177.6 ± 17.3 mV for chemical oxygen demand (COD) of 100 mg/L to 524.7 ± 3.2 mV for COD of 400 mg/L, in response to the decrease in anodic potential from −110.5 ± 21.6 mV to −508.7 ± 6.9 mV (Figure 1a and b). The results indicated that organic matters produced from the hydrolysate could be easily utilized by anodic microorganism and release electrons, decreasing the anodic potential and consequently increasing the cell voltage [14]. The stable anodic potential was appropriately −300 mV (versus standard hydrogen electrode), similar to that of −340 mV observed by Wang *et al*. [15]. For relatively low COD concentrations (100 and 150 mg/L), the cell voltage showed a large difference between two separate cycles; while the COD concentration increased to 200 mg/L, the voltage was well reproduced, suggesting that the saturated COD for voltage output was 200 mg/L. The saturated COD concentration in the present study was far lower than that of the 1,000 mg/L observed when wheat straw hydrolysate was used as fuel in a dual-chamber MFC [16]. A lower saturated COD concentration indicated higher capacity of power production from the fuel based on the same quantity. Furthermore, the discharge time increased from 17.1 ± 1.2 hours to 49.8 ± 2.4 hours relying on the increased COD concentration.

**Figure 2:**
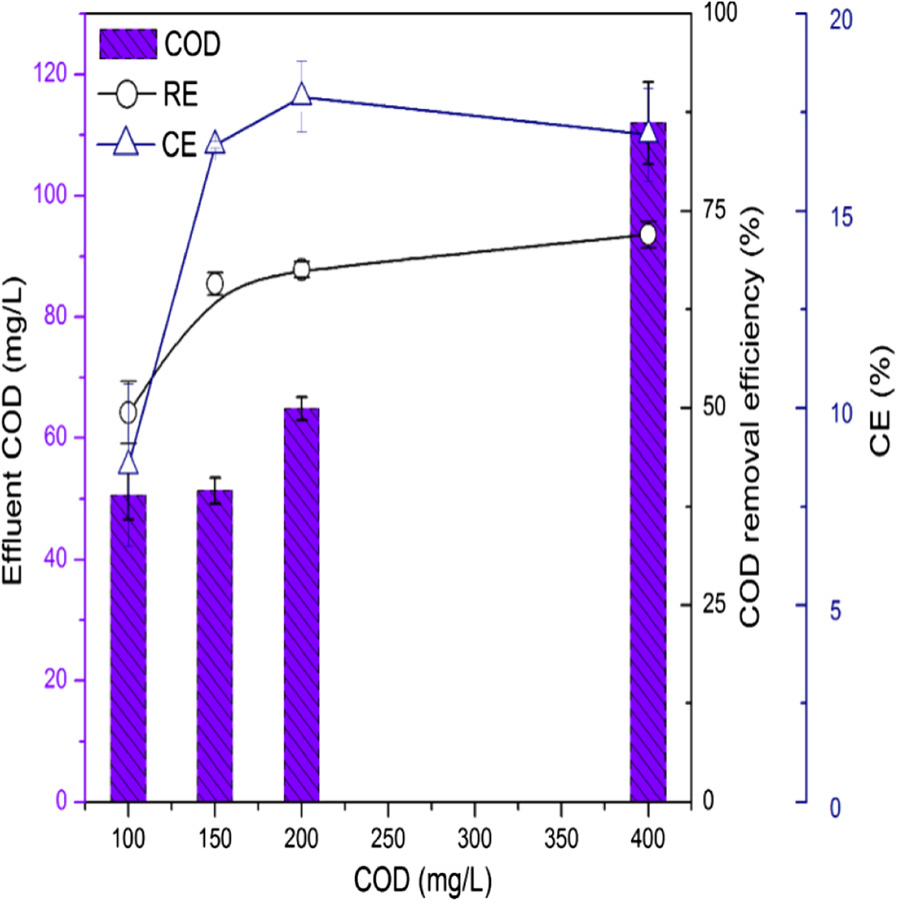
**Effluent chemical oxygen demand (COD) concentration and COD removal efficiency (RE) and coulombic efficiency (CE).** The initial COD concentration was 100, 150, 200, and 400 mg/L with an external resistance of 500 Ω.

Coulombic efficiency (CE) indicates the ratio of total electrons recovered as electric current from organic matter. In this study, the CE was calculated in the range from 8.5% to 17.9%, and the COD removal efficiency ranged from 49.4 ± 4.0% to 72.0 ± 1.7% (Figure 2). The present CE was lower than that of wheat straw- [16] or corn stover biomass-fuelled [17] MFCs, which were 15.5% to 37.1% and 19.3% to 25.6%, respectively; whereas it was higher than that previously reported using real wastewater as fuels, which for example, is less than 1% for fermented wastewater [18], and a maximum CE of 8% for starch processing wastewater [19]. For air-cathode MFCs, oxygen diffused to the anode chamber can aerobically consume substrates other than anaerobically generated electrons, which would be the reason for the low CE of air-cathode MFCs [20].

**Figure 3:**
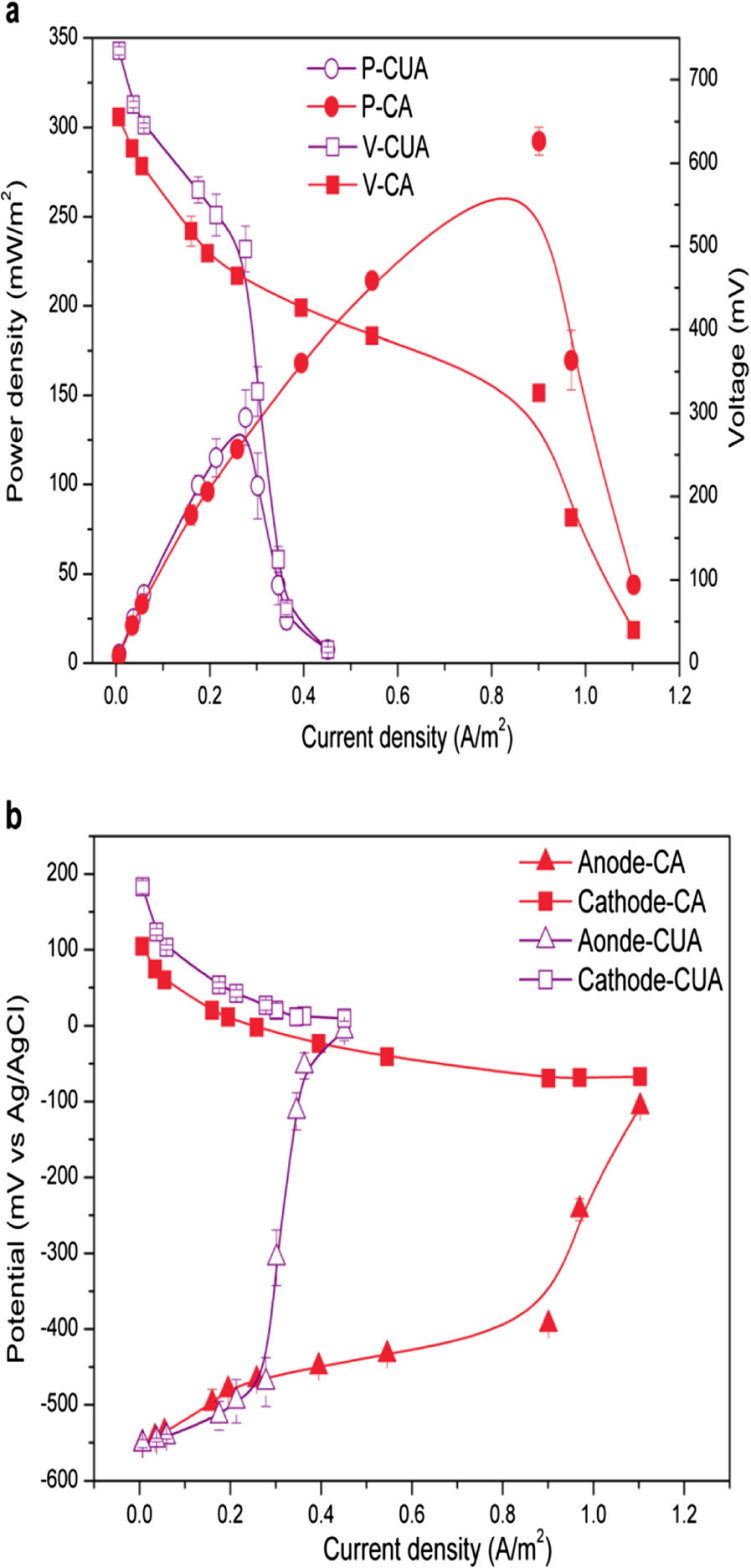
**Power density and electrode potentials as a function of current density. (a)** Polarization curves, and **(b)** electrode potentials for conductivity unadjusted (CUA) and adjusted (CA) solutions with hydrolysate of a COD concentration of 400 mg/L. The conductivity was adjusted from 5.6 mS/cm to 17 mS/cm with addition of NaCl.

The maximum power density (P_max_) was determined as 137.6 ± 15.5 mW/m^2^ for COD of 400 mg/L, at a current density of 0.28 A/m^2^ (Figure 3a). It was further promoted to 293.3 ± 7.9 mW/m^2^ at a current density of 0.90 A/m^2^ when the solution conductivity was adjusted from 5.6 mS/cm to 17 mS/cm through addition of NaCl. Consequently, the internal resistance (R_int_) of the MFC was decreased from 714.4 ± 19.1Ω to 229.9 ± 14.5Ω. Moreover, conductivity adjustment enhanced the anodic performance, relieving the mass transfer limitation while limiting the cathode performance (Figure 3b).

The P_max_ (293.33 ± 7.89 mW/m^2^) was larger than that of wheat straw hydrolysate (123 mW/m^2^). Based on the maximum power of 1.08 mW (293.33 mW/m^2^) and the stable discharge time of 24 hours, 2.592 × 10^-5^ kWh of electric power can be extracted from a COD concentration of 400 mg/L. The worldwide generation rate of rice straw was about 731 million dry tons in 2007. Therefore, a total amount of 1.51 × 10^18^ mg/L COD can be produced, from which 9.78 × 10^10^ kWh of electric power can be extracted. In 2007, the worldwide per capita consumption of electric power was 2,752 kWh (http://www.chinaero.com.cn). Therefore, the electric power extracted from rice straw can meet the annual demand of 35.56 million people for electric power.

Some factors, including reactor design, distance between electrodes, solution conductivity, and electrode material, can affect the performance of MFCs. In the present study, a graphite brush anode with a large specific surface area for attachment of exoelectrogens was adopted, which was previously reported as very beneficial for power output [21]; whereas on the other hand, it is difficult to control the distance between electrodes with the cylindrical type of brush anode, because electrode distance has been verified as an important factor capable of affecting the R_int_ of MFCs [22]. The R_int_ of MFCs can be separated into that of the anode, electrolyte, and cathode. The conductivity of the electrolyte can affect the ion transport within the electrolyte. Conductivity adjustment increased the mass transfer rate of organic matters to the anodic biofilm, providing sufficient electron donors and thus, improved the performance of the anodic exoelectrogens. On the other hand, the addition of NaCl to the electrolyte reduced the performance of the cathode. It has been reported that Cl^-^ can cause deterioration in the performance of Pt towards the oxygen reduction reaction due to the strong interaction between adsorbed Cl^-^ and Pt, which could suppress the adsorption of O_2_ and the formation of Pt site-pairs acquired for breaking the O-O band [23]. Moreover, increasing the solution conductivity could promote the ionic transfer rate and thus decrease the solution resistance [24]. Therefore, the overall effects of NaCl on the anode and electrolyte exceeded that of the cathode, reducing the overall R_int_ of the MFC and consequently promoting the P_max_ output.

### Microbial richness and diversity

**Figure 4:**
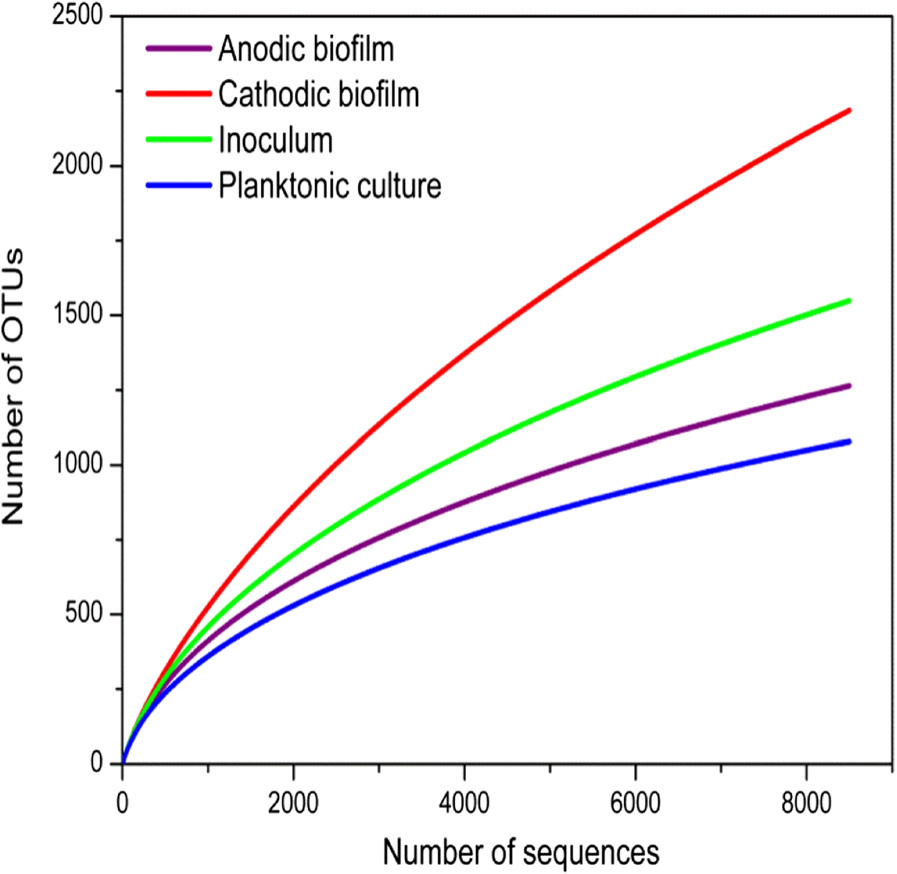
**Rarefaction curves based on pyrosequencing of bacterial communities.** Samples were collected from anodic and cathodic samples, and planktonic culture. Inoculum was also analyzed to observe the change in microbial structure after microbial fuel cell operation. The operational taxonomic units (OTUs) were defined at 0.03 distances.

Four 16S rRNA gene libraries were constructed for the 454-pyrosequencing. The qualified sequences for the microbial samples were 8,615 to 14,308 (Figure 4). The sequences were clustered to represent operational taxonomic units (OTUs) with 3% nucleotide dissimilarity. To reduce sequencing efforts to compare the number of OTUs exactly, 8,500 sequences were randomly selected to calculate the number of OTUs in each sample. The total observed OTUs were 1,242, 2,200, 1,549 and 1,085 for anodic and cathodic biofilm, inoculums, and planktonic culture, respectively (Additional file 1: Table S1). The results illustrated that MFC operation increased the microbial richness of the cathodic biofilm, whereas the richness of the anodic biofilm and planktonic culture were reduced. The Shannon index provides information on species richness and diversity [25]. The cathodic biofilm showed the largest Shannon index (6.5), followed by the inoculum (6.2), anodic biofilm (5.9), and the planktonic culture (5.7). The Shannon index further confirmed that the operation reduced the microbial diversity of the anodic biofilm, which might be due to the selection of exoelectrogens caused by the generation of electricity [26]. However, the microbial diversity of the cathodic biofilm was increased compared to the inoculum. In an air-cathode MFC, ambient air transfers through the diffusion layer into the anolyte; however, the formation of cathodic biofilm decreases the transfer rate of air into the anolyte, preventing mass transfer of organic matter, and the transport of OH^–^ out of the biofilm [27]. Therefore, the formation of cathodic biofilm resulted in respiratory environments with different levels of oxygen, organic matter concentration, and pH. This could be the reason why there was an increase in microbial diversity in the cathodic biofilm. In an air-diffusion biocathode MFC, however, the microbial diversity in cathodic biofilm was smaller than that in planktonic culture [28], possibly due to the different MFC configuration and carbon source.

### Microbial community analysis

**Figure 5:**
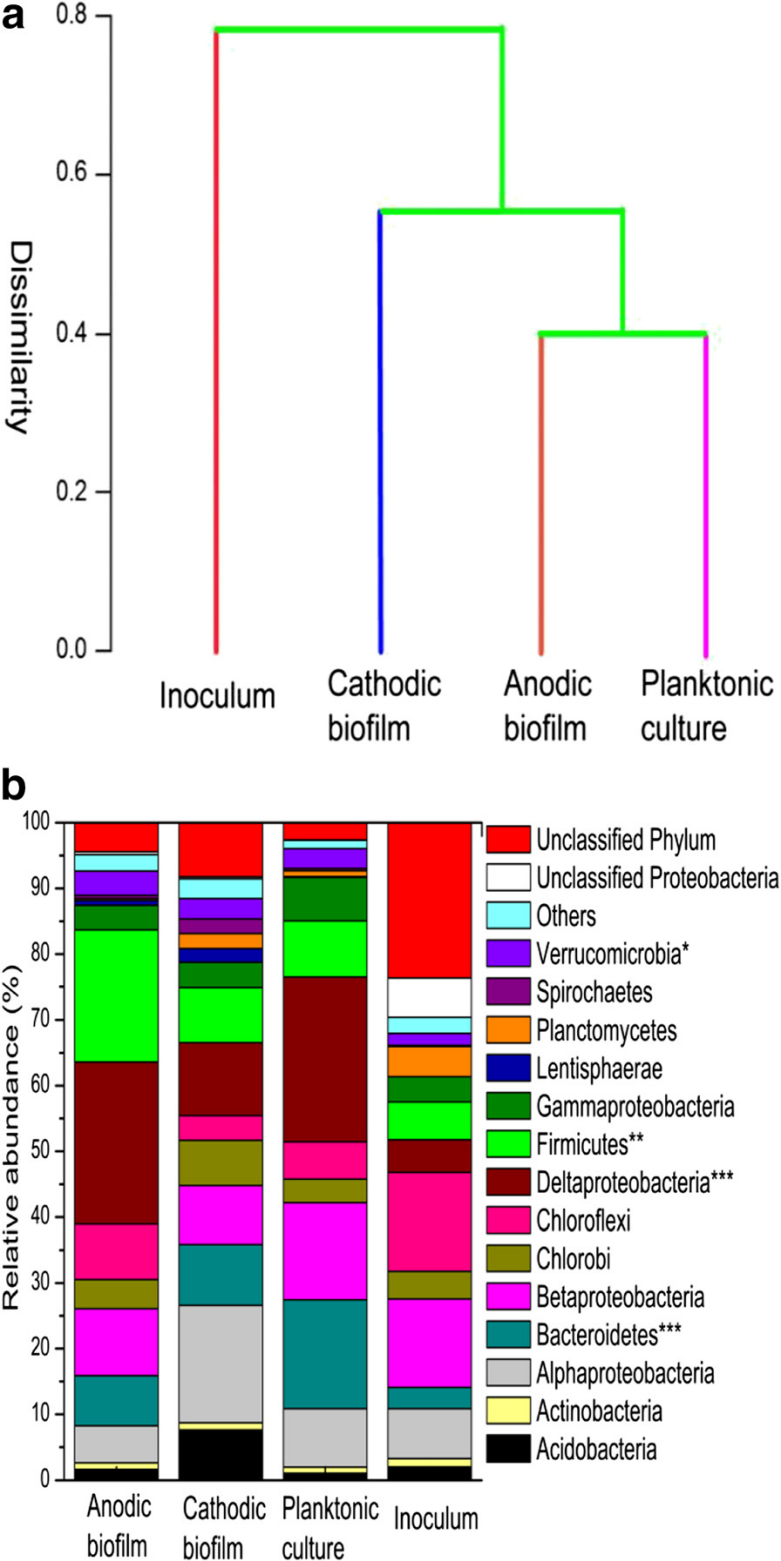
**Microbial community analysis of the collected microbial samples from anodic and cathodic biofilm, planktonic culture, and inoculum. (a)** Jaccard clustering results of bacterial communities defined at 0.03 distances, and **(b)** microbial community composition at phylum level. Phyla accounting for less than 1% of the total composition in all four libraries were classified as others. *P <0.05, **P <0.01, ***P <0.001.

A taxonomic supervised dendrogram was prepared to examine the overall variation in the microbial community of the samples. As demonstrated in Figure 5a, the anodic biofilm and planktonic culture were relatively well-clustered, and the cathodic biofilm was clustered closer to the inoculum in comparison to the anodic and planktonic culture. The results indicated that the electrode reactions and niches could influence the microbial community structure which was significantly differentiated from that of the inoculum.

Figure 5b shows the relative abundance of the four microbial communities at phylum level. *Proteobacteria* including α-, β-, δ- and γ- was the predominant phylum accounting for 44.2% , 41.9%, 55.2% and 29.8% of the total abundance in the anodic and cathodic biofilm, planktonic culture and inoculum, respectively. Among the *Proteobacteria*, the δ- class showed a great increase from 5.0% in the inoculum to 11.1% to 25.0% in the MFC samples; the α- class increased from 7.5% in the inoculum to 8.8% in the planktonic culture, and 17.9% in the cathodic biofilm, whereas it decreased to 5.6% in the anodic biofilm. Moreover, *Bacteroidetes* increased from 3.3% in the inoculum to 7.7% in the anodic biofilm, 9.2% in the cathodic biofilm, and 16.6% in the planktonic culture; *Firmicutes* were enriched from 5.8% in the inoculum to 20.1%, 8.3%, and 8.6% in the anodic biofilm, cathodic biofilm and planktonic culture, respectively. *Bacteroidetes* and *Firmicutes* made up the subdominant members accounting for 27.7% in the anodic biofilm, 17.5% in the cathodic biofilm, and 25.2% in the planktonic culture, respectively. *Chloroflexi* was reduced after MFC operation from 15.0% in the inoculum to 8.4%, 3.8%, and 5.7% in the anodic biofilm, cathodic biofilm, and the planktonic culture, respectively. Furthermore, unclassified phylum was significantly reduced to 2.6% to 8.3% in the MFC samples from 23.7% in the inoculum. As previously reported, the species, such as *Shewanella putrefacies* IR-1 [29], *Geobacter sulfurreducens*[30], and *Ochrobacrum anthropi* YZ-1 [31] belonging to *Proteobacteria* were the most important exoelectrogens in the anodic biofilm. Moreover, a few isolated exoelectrogens belonging to *Firmicutes* such as *Clostridium butyricum* EG3 [32], *Desulfitobacterium hafniense* strain DCB2 [33], *Thermincola sp.* strain JR [34], and *Thermincola ferriacetica*[35], were known as a source of exoelectrogens. The present results differed from that of the observed exoelectrogens of a two-chambered MFC using wheat straw biomass for which the predominant culture was *Bacteroidetes* with 40% of sequences [16]. And *Bacteroidetes* and γ-*proteobacteria* were the most abundant phylum in the anodic biofilm of an air-cathode dual-chamber MFC fed with glucose and glutamate [36]. In MFCs, the microbial community was greatly influenced by the substrates, operation time, and architecture of the cell [16,37,38]. Rice straw hydrolyte generally consists of glucose, xylose, arabinose, acetic acid, and small amount of furfural and 5-hydroxymethyl-furfural [39]. The component of rice straw hydrolyte and single-chamber design in the present study is proposed the factor resulting in different microbial community to the previous studies.

*Proteobacteria* (41.9%, α-17.9%, δ-11.1%, β-9.0%, and γ-3.8%), *Bacteroidetes* (9.2%), *Firmicutes* (8.3%), *Acidobacteria* (7.6%), and *Chlorobi* (6.9%) made up the dominant groups of the cathodic biofilm in this study. In a two-chamber air-diffusion biocathode MFC, the dominant groups were observed as *Proteobacteria* (39.9%, α- 31.7%, γ- 3.8%, β- 2.5%, and δ- 1.1%), *Planctomycetes* (29.9%) and *Bacteroidetes* (13.3%) [28]. The cathodic biofilm in an air-cathode single-chamber MFC can utilize organic carbon sources whereas the cathodic biofilm in a two-chamber air-diffusion MFC was fed with an inorganic carbon source, such as NaHCO_3_. This should be the reason for the different dominant groups in the two cathodic biofilms.

**Figure 6:**
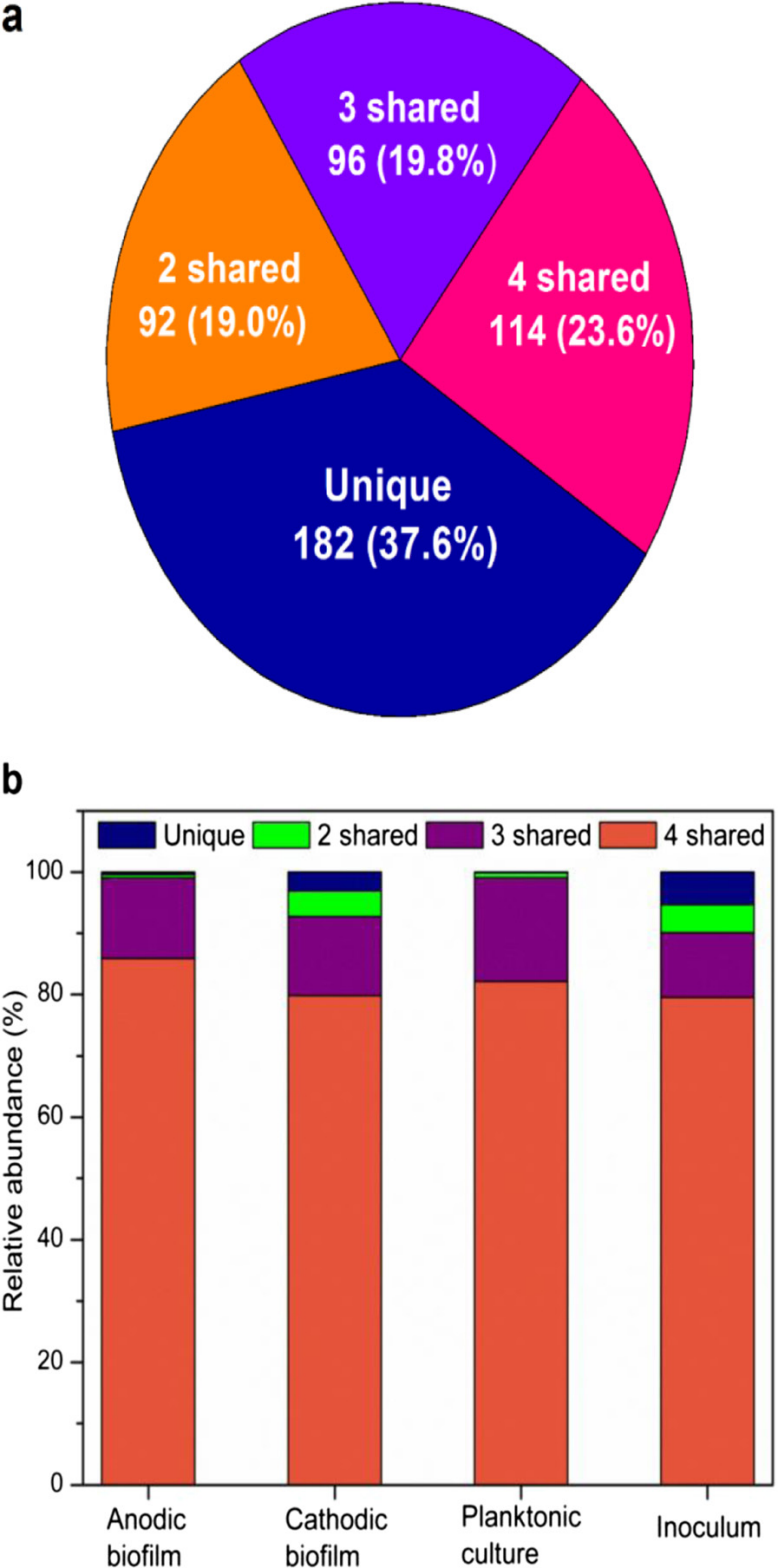
**Distribution of detected genus in the samples. (a)** Overlapping microbial communities of anodic and cathodic biofilm, planktonic culture and inoculum at genus level, and **(b)** ratio of shared genus to the total genus of different samples.

A total of 484 genera were obtained using the Ribosomal Database Project (RDP) classifier, of which 114 genera were commonly shared by all samples (Figure 6a). They accounted for 85.9%, 79.8%, 82.1% and 79.5% of the anodic and cathodic biofilm, planktonic culture and inoculum, respectively (Figure 6b). There were 182 genera that appeared in only one sample, accounting for 0.4%, 3.1%, 0.1% and 5.3% of the classified sequences in the anodic and cathodic biofilm, planktonic culture and inoculum, respectively. The results suggested that the differentiation of microbial community structure in the samples was caused by a minor portion of the genus.

### Exoelectrogens

Exoelectrogens play a key role in the generation of electric power through transferring electrons produced from organic matters to the surface of anode materials. Exoelectrogens observed in the present study with greater than 1% abundance are summarized in Table 1. In anodic biofilm, five genera of known exoelectrogens accounted for 23.5% in total communities, including *Desulfobulbus* (11.0%), *Geobacter* (5.3%), *Desulfovibrio* (3.6%), *Pseudononas* (2.3%), and *Comamonas* (1.4%). The genera of exoelectrogens in planktonic culture included *Desulfoblbus* (19.0%), *Desulfovibrio* (4.5%), *Pseudomonas* (2.9%), and *Comamonas* (2.2%), accounting for 28.5% of the total genus. For the cathodic biofilm, however, exoelectrogen species was reduced to three genera, including *Desulfoblbus* (7.8%), *Comamonas* (1.9%), and *Desulfovibrio* (1.3%), with a total abundance of 11.0%. In the inoculum, only one genus with an abundance greater than 1% (*Clostridium*, 1.6%) was discovered, demonstrating that the electricity production can influence the richness of exoelectrogens in MFCs. On the other hand, *Clostridium* was significantly reduced after MFC operation, indicating that the genus of *Clostridium* lost the competition of electron generation compared to other exoelectrogens. The genus of *Desulfobulbus* was the common predominant exoelectrogen in anodic and cathodic biofilm, and planktonic culture, demonstrating that this genus was well adopted in the operating conditions and might attribute to the power generation as a main exoelectrogen in the electrodes. *Pseudomonas* was shared in the anodic biofilm and planktonic culture, but not in the cathodic biofilm, indicating that *Pseudomonas* was not suitable to utilize external electron transferred from the anode. *Geobacter*, an important genus attributing to the power generation in an MFC [40,41], was only dominant in the anodic biofilm. Among the *Geobacter* species, *G. sulfurreducens* and *G. metallireducens*, which were revealed to transfer electrons through nanowire to electron acceptors [42], accounted for 2.9% and 0.2% of the total abundance, respectively. These results suggested the attribution of *Geobacter* species to current generation in the present study. In MFCs, an opposite electron flow of the anode and cathode reaction occurred, and the physical and chemical environment in the anodic and cathodic biofilm was also different. This might be the reason for the different predominant exoelectrogens observed in the anodic and cathodic biofilm.

**Table 1:** Exoelectrogenic genus observed in different MFC samples and the inoculum (>1%)

**Genus**	**Anodic**	**Planktonic**	**Cathodic**	**Inoculum, %**
	**biofilm, %**	**culture, %**	**biofilm, %**	
*Clostridium*	-	-	-	1.6
*Comamonas*	1.4	2.2	1.9	-
*Desulfobulbus*	11.0	19.0	7.8	-
*Desulfovibrio*	3.6	4.5	1.3	-
*Geobacter*	5.3	-	-	-
*Pseudomonas*	2.3	2.9	-	-

### Sulfate reducing bacteria

The diluted-acid treatment of rice straw produced sulfate as a main component of the rice straw hydrolysate. The sulfate-reducing bacteria (SRB) could serve a main role in sulfate reduction in this study. Twenty genera of putative sulfate and sulfur-reducing bacteria were detected, and belonged to δ-*proteobacteria* and *Firmicutes*. The dominant members were *Desulfobulbus*, *Desulfovibrio, Desulfomicobium*, *Desulforhabdus* and *Geobacter* (Additional file 1: Table S2), of which *Desulfobulbus*, *Desulfovibrio* and *Geobacter* are well-known to play an important role in transferring electrons to the anode. The abundance of SRB was remarkably increased in the three MFC samples compared with the inoculum community. The SRB from the anodic biofilm and planktonic culture were approximately three times more abundant than in the cathodic biofilm. A relatively low abundance of SRB in the cathodic biofilm can be explained by the diffused oxygen to the cathodic biofilm which might have competed with sulfate as an electron acceptor. Interestingly, a considerably higher number of SRB such as *Desulfobulbus* and *Desulfovibrio* were observed in the planktonic culture than in the anodic biofilm. The results showed opposite trends in other dominant groups such as *Desulfomicobium*, *Desulforhabdus*, and *Geobacter*, representing greater abundance in the anodic biofilm than in the planktonic culture. Although SRB was reported to play an important role in transferring electrons on the anode, some groups of SRB that could not form biofilm on the electrode may also reduce sulfate in the planktonic niche [42]. It has already been reported that SRB demonstrate functional dynamics, including electron transfer, sulfate reduction, and converting organic matters, such as acetic and butyric acids to alcohols and acetone via direct electron transfer [43-45]. In the present study, therefore, high-abundance groups (*Desulfobulbus* and *Desulfovibrio*) may perform both electron transfer on the electrode and sulfate reduction in the electrolyte, whereas low-abundance groups may focus primarily on transferring electrons in the electrode.

## Conclusions

The present study confirmed that rice straw hydrolysate is flexible as fuel for MFCs to generate electric power. The microbial community was differentiated after the MFC operation. Furthermore, the differentiation in the microbial community structure resulted from a small portion of the genus. The microbial community of the anodic biofilm had a similar microbial structure to the planktonic culture, but it was different to the cathodic biofilm. The known exoelectrogens were differently enriched depending on the niches caused by the different electrode reaction and respiratory environment. Sulfate-reducing bacteria were greatly abundant due to sulfate production by the dilute-acid treatment of the rice straw. They might play different roles in different niches.

## Materials and methods

### Rice straw hydrolysate preparation

The rice straw collected from Daejeon, Korea was rinsed with tap water and then distilled water, and further dried in an oven at 50°C. The dried rice straw was milled with a juice extractor and was then hydrolyzed using the diluted-acid method (1:10, w/v) [46]. The hydrolysate was further treated by an over-liming process as previously reported to reduce toxic inhibitors [47]. The pH of the resultant hydrolysate was adjusted to approximately 7.0 with concentrated H_2_SO_4_, and the residue was separated by centrifugation (3,000 rpm, 3 minutes). The COD concentration of the resultant hydrolysate was 20,689 mg/L.

### MFC fabrication

The MFC reactor was made of Plexi-glass (6 cm × 6 cm × 6.5 cm), with 220 mL of working volume. The carbon brush anode (length 2 cm, diameter 3 cm) was prepared by twisting carbon fiber (PANEX® 35, Zoltek) with stainless steel wire. The cathode was a commercially available ELAT® gas diffusion electrode (Lot #LT 120E-W: 090205) with Pt catalyst (20%). The catalyst side of the cathode was coated with Nafion® polymer dispersion (5%, Aldrich) and dried in air, leading to a Nafion loading rate of 0.5 mg/cm^2^. The effective area of the cathode was 36 cm^2^. To determine the anodic potential, an Ag/AgCl (3.3 M KCl) was introduced to the electrolyte.

### Culture inoculation and MFC operation

To start up the MFC, 10 mL anaerobic sludge collected from the anaerobic digester of a wastewater treatment plant in Orgchen, Korea was mixed with 210 mL medium solution and pumped into the MFC. The medium solution contained 0.1 g/L KCl, 0.5 g/L NH_4_Cl, 0.1 g/L MgCl_2_, 0.1 g/L CaCl_2_, 0.3 g/L KH_2_PO_4_, 0.5 g/L NaHCO_3_, and 1.36 g/L CH_3_COONa · 3H_2_O as an electron donor. Each time the voltage at a fixed external resistance of 500 Ω dropped below 35 mV, sodium acetate was added to the solution until there was a repeatable voltage output. After the cell was successfully started up, the solution was switched to rice straw hydrolysate which was diluted to different COD concentrations (100, 150, 200, and 400 mg/L) with distilled water and then buffered with 1.05 g/L NH_4_Cl, 1.5 g/L KH_2_PO_4_, and 2.2 g/L K_2_HPO_4_. All the experiments were performed by batch mode and the solution was stirred with a magnetic stirring bar. Temperature was controlled at 30 ± 1°C in an incubator.

### Analysis and calculation

COD was analyzed following the standard method of Korea after filtering through a glass filter paper to remove bacteria. Voltage (V) was monitored using a LabView program every ten minutes. Power density was normalized to the cathode surface area (A) as follows:

$$P=V^{2}/R\cdot A,$$

and current density (j) was calculated as follows:

j=V/R·A,

where R is the external resistance. In order to determine the P_max_, the external resistance was varied between 30 k and 10 Ω. To observe the effect of conductivity on the performance of the MFC, the conductivity of the electrolyte was adjusted from 5.6 mS/cm to 17 mS/cm using NaCl. The CE was calculated as:

$$\mathrm{CE}=C_{i}/C_{o},$$

where C_i_ is the coulomb collected from the passed current and C_o_ is the coulomb generated from the consumed organic matters. R_int_ was determined as the slope of the i-V curve according to:

$$V=U_{\mathrm{cell}}-iR_{\mathrm{int}},$$

where U_cell_ is the electromotive force of the cell [15].

### Microbial analysis

#### DNA extraction, PCR, and FLX titanium pyrosequencing

Samples were collected from inoculum sludge, anodic and cathodic biofilm, and planktonic culture for microbial analysis. DNA was extracted using PowerSoil™ DNA Isolation Kit (MoBio, Carlsbad, CA, USA) following the manufacturer’s instructions. The following universal 16S rRNA primers were used for the PCR reactions: F563 (AYTGGGYDTAAAGNG) and BSR926 (CCGTCAATTYYTTTRAGTTT). Barcode sequences (AGCATCTG, AGCATGAG, AGCTCAGC and AGCTCATG for anodic biofilm, cathodic biofilm, planktonic culture, and inoculum, respectively) were attached between the 454 adaptor sequence and the forward primers. Each PCR reaction was carried out with two of the 25-μl reaction mixtures containing 60 ng of DNA, 10 μM of each primer (Macrogen, Seoul, Korea), and AccuPrime™ *Taq* DNA Polymerase High Fidelity (Invitrogen, Madison, WI, USA) in order to obtain the following final concentrations: 1.25 U of *Taq* polymerase, 50 mM of MgSO_4_, and 10× of the PCR buffer. A C1000TM thermal cycler (Bio-rad, Hercules, CA, USA) was used for the PCR as follows: (i) an initial denaturation step at 94°C for 3 minutes, (ii) 30 cycles of annealing and extending (each cycle occurred at 95°C for 60 s followed by 55°C for 45 s and an extension step at 72°C for 60 s), and (iii) the final extension at 72°C for 5 minutes. After this PCR amplification, the amplicons were purified by one-time gel electrophoresis/isolation and two-time purifications using a QIAquick Gel extraction kit (Qiagen, Valencia, CA, USA) and QIAquick PCR purification kit (Qiagen). In order to recover a sufficient amount of purified amplicons from the purification steps, two 25-μl reaction mixtures were combined into one prior to amplicon purification. All four amplicons were pooled and amplicon pyrosequencing was performed by Macrogen Inc. (Seoul, South Korea) using a 454/Roche GS-FLX titanium instrument (Roche, Nutley, NJ, USA).

### Microbial community and classification

Sequences were analyzed following the modified protocol using Mothur. The range of flow was modified from 450 to 720 to obtain highly accurate sequences. Chimera sequences were removed by the Uchime algorithm with self-references. Filtered sequences were aligned to Silva Gold aligned sequences, and clustered with the furthest algorithm at 0.03 distances. Sequences were classified using the RDP training set (Version 9) with a threshold of 50%. Classified sequences were analyzed into phylotype at phylum and genus level. To reduce sequencing efforts from samples, the smallest sequence numbers were selected to measure the alpha diversity, such as observed OTUs, the Shannon index, and rarefaction curves. A taxonomy-supervised dendrogram was constructed to compare microbial communities from samples using relative abundances. After calculating the relative abundance of genus including unclassified sequences at genus level, a distance matrix (vegdist) was produced and clustered through the average algorithm (hclust) using Vegan package from R.

## Abbreviations

CE: Coulombic efficiency; COD: Chemical oxygen demand; MFC: Microbial fuel cell; OTU: Operational taxonomic unit; Pmax: maximum power density; RDP: Ribosomal Database Project; Rint: internal resistance; SRB: Sulfate-reducing bacteria.

## Competing interests

The authors declare that they have no competing interests.

## Authors’ contributions

ZW designed the research and performed the MFC operation. TL carried out the microbial community analysis and prepared the related part of manuscript. ZW prepared the manuscript. BL, CC and JP reviewed the manuscript. All authors read and approved the final manuscript.

## Supplementary Material

Additional file 1 **Alpha diversity of, and sulfate-reducing bacteria in the microbial samples. Table S1:** Summary of alpha diversity of microbial communities in anodic and cathodic biofilms, planktonic culture, and inoculum. **Table S2:** Distrubution of sulfate and sulfur-reducing bacteria observed in anodic and cathodic biofilms, planktonic culture, and inoculum (%). Click here for file
