# Supplementary material for: Microbial community structures differentiated in a single-chamber air-cathode microbial fuel cell fueled with rice straw hydrolysate
Source: Biotechnol Biofuels. 2014 Jan 17;7:9. doi: 10.1186/1754-6834-7-9 (PMC3896841; doi:10.1186/1754-6834-7-9)
Supplement: Additional file 1 — Alpha diversity of, and sulfate-reducing bacteria in the microbial samples. Table S1: Summary of alpha diversity of microbial communities in anodic and cathodic biofilms, planktonic culture, and inoculum. Table S2: Distrubution of sulfate and sulfur-reducing bacteria observed in anodic and cathodic biofilms, planktonic culture, and inoculum (%). [file 1754-6834-7-9-S1.pdf]

Table S1 Summary of alpha diversity of microbial communities in anodic and cathodic biofilms, planktonic culture, and inoculum

Sample	Sequences	Subsample	Observed OTUs	Shannon Index	Evenness
Anodic biofilm	11,586	8,500	1,242 ± 14	5.9	0.83
Cathodic biofilm	14,308	8,500	2,200 ± 25	6.5	0.85
Inoculum	8,615	8,500	1,549 ± 2	6.2	0.84
Planktonic culture	8,683	8,500	1,085 ± 4	5.7	0.82

Table S2 Distribution of sulfate and sulfur reducing bacteria observed in anodic and cathodic biofilms, planktonic culture, and inoculum (%)

Genus	Anodic biofilm	Cathodic biofilm	Planktonic culture	Inoculum
Desulfobulbus	11.00	7.84	18.96	0.03
Desulfomicrobium	2.68	0.31	0.55	0.21
Desulforhabdus	1.70	0.03	0.50	0.23
Desulfovibrio	3.55	1.33	4.45	0.05
Geobacter	5.29	0.18	0.24	0.01
The others	0.35	0.20	0.14	1.18
Total	24.57	9.89	24.83	1.72
